# Predicting three or more metastatic nodes using contrast-enhanced lymphatic US findings in early breast cancer

**DOI:** 10.1186/s13244-024-01648-1

**Published:** 2024-03-25

**Authors:** Zihan Niu, Yunxia Hao, Yuanjing Gao, Jing Zhang, Mengsu Xiao, Feng Mao, Yidong Zhou, Ligang Cui, Yuxin Jiang, Qingli Zhu

**Affiliations:** 1 Department of Ultrasound, Peking Union Medical College, Peking Union Medical College Hospital, Chinese Academy of Medical Sciences and Peking Union Medical College, Beijing, 100730 People’s Republic of China; 2 Department of Ultrasound, Peking University Third Hospital, Haidian District, 49 North Garden Road, Beijing, 100191 People’s Republic of China; 3 Department of Breast Surgery, Peking Union Medical College Hospital, Peking Union Medical College, Chinese Academy of Medical Sciences, No. 1 Shuaifuyuan, Dongcheng District, Beijing, 100730 People’s Republic of China

**Keywords:** Breast neoplasms, Contrast media, Lymphatic metastasis, Ultrasonography

## Abstract

**Objectives:**

To develop and validate a nomogram for predicting ≥ 3 metastatic axillary lymph nodes (ALNs) in early breast cancer with no palpable axillary adenopathy by clinicopathologic data, contrast-enhanced (CE) lymphatic ultrasound (US), and grayscale findings of sentinel lymph nodes (SLNs).

**Materials and methods:**

Women with T1-2N0 invasive breast cancer were consecutively recruited for the CE lymphatic US. Patients from Center 1 were grouped into development and internal validation cohorts at a ratio of 2:1. The external validation cohort was constructed from Center 2. The clinicopathologic data and US findings of SLNs were analyzed. A nomogram was developed to predict women with ≥ 3 metastatic ALNs. Nomogram performance was assessed with the area under the receiver operating characteristic curve (AUC) and calibration curve analysis.

**Results:**

One hundred seventy-nine from Center 1 were considered the development cohorts. The remaining 90 participants from Center 1 were internal cohorts and 197 participants from Center 2 were external validation cohorts. The US findings of no enhancement (odds ratio (OR), 15.3; *p* = 0.01), diffuse (OR, 19.1; *p* = 0.01) or focal eccentric (OR, 27.7; *p* = 0.003) cortical thickening, and absent hilum (OR, 169.7; *p* < 0.001) were independently associated with ≥ 3 metastatic ALNs. Compared to grayscale US or CE lymphatic US alone, the nomogram showed the highest AUC of 0.88 (0.85, 0.91). The nomogram showed a calibration slope of 1.0 (*p* = 0.80–0.81; Brier = 0.066–0.067) in validation cohorts in predicting ≥ 3 metastatic ALNs.

**Conclusion:**

Patients likely to have ≥ 3 metastatic ALNs were identified by combining the lymphatic and grayscale US findings of SLNs. Our nomogram could aid in multidisciplinary treatment decision-making.

**Trial registration:**

This trial is registered on www.chictr.org.cn: ChiCTR2000031231. Registered March 25, 2020.

**Critical relevance statement:**

A nomogram combining lymphatic CEUS and grayscale US findings of SLNs could identify early breast cancer patients with low or high axillary tumor burden preoperatively, which is more applicable to the Z0011 era. Our nomogram could be useful in aiding multidisciplinary treatment decision-making for patients with early breast cancer.

**Key points:**

• CEUS can help identify and diagnose SLN in early breast cancer preoperatively.

• Combining lymphatic and grayscale US findings can predict axillary tumor burden.

• The nomogram showed a high diagnostic value in validation cohorts.

**Graphical Abstract:**

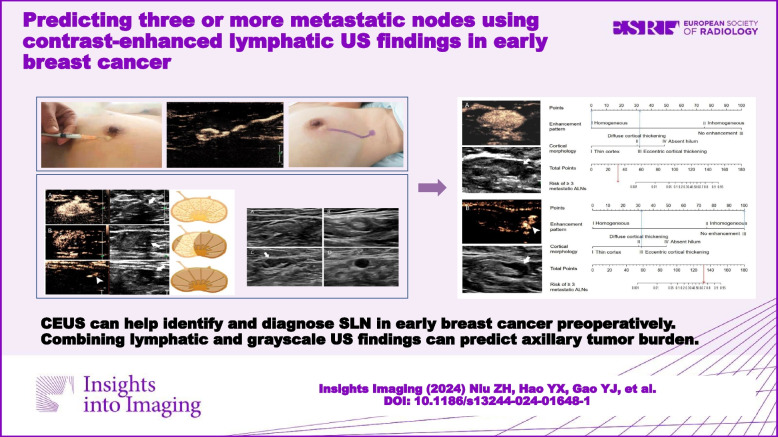

**Supplementary Information:**

The online version contains supplementary material available at 10.1186/s13244-024-01648-1.

## Introduction

Axillary lymph node (ALN) metastasis is the most important predictor of breast cancer prognosis [1]. Sentinel lymph node (SLN) biopsy is the standard surgical procedure for early breast cancer with preoperative negative ALN [2]. The American College of Surgeons Oncology Group (ACOSOG) Z0011 study found that for T1–2 breast cancer patients with no clinically palpable axillary adenopathy, the complete dissection of ALNs is not warranted in women with fewer than three involved ALNs who undergo breast-conserving surgery and whole-breast radiotherapy [3]. Among all clinically node-negative patients, approximately 25–35% harbor nodal metastases, while approximately 10% have ≥ 3 metastatic ALNs [4–7]. Preoperative identification of ≥ 3 metastatic ALNs can be an indication of neoadjuvant systemic treatment or direct ALN dissection (ALND) [7, 8].

Ultrasound (US) is the primary method for evaluating the axilla in newly diagnosed breast cancer patients [9]. Preoperative US-guided biopsy helps the clinician to determine whether the patients have axillary metastasis, but it cannot accurately identify high metastatic ALNs [10]. Contrast-enhanced (CE) lymphatic ultrasound (US) can be used for detecting sentinel lymph nodes in cancer patients. The SLN can be traced via the accumulation of contrast agents in the enhanced lymphatic channels. Previous studies have demonstrated that the SLN located by CE lymphatic US is the same SLN or one of the SLNs located by methylene blue [11–15]. CE lymphatic US is confirmed as a safe and effective preoperative SLN identification method [11–16].

How to stratify early breast cancer patients with ≥ 3 metastatic ALNs is the question in the Z0011 era. CE lymphatic US enhancement pattern has been shown to be helpful in detecting metastasizing SLNs, but the diagnostic value of enhancement patterns yielded an unsatisfactory result with a specificity of 34.2% and an accuracy of 37.9% for ≥ 3 metastatic ALNs [17]. Given this fact, grayscale US could help to further improve the evaluation of metastatic ALNs, but the combination of enhancement patterns and grayscale US findings of SLNs for predicting ≥ 3 LN metastases has not yet been well established. In addition, the axillary metastatic status seems to be usually related to the clinical characteristics and pathologic features of the primary tumor [18]. Therefore, the purpose of our study was to construct a nomogram to preoperatively predict ≥ 3 metastatic ALNs in early breast cancer based on clinicopathologic data, CE lymphatic US, and grayscale US findings of SLNs.

## Materials and methods

This was a prospective multicenter study. This study was approved by the Institutional Review Board. All participants signed consent forms.

### Study cohorts

Women with newly diagnosed clinical T1–2 invasive breast cancer and no palpable ALN (cN0) were consecutively recruited for axillary lymphatic US evaluation. Exclusion criteria are as follows: (i) allergic to US contrast agent, (ii) history of ipsilateral breast cancer with axillary surgery or radiotherapy, (iii) received neoadjuvant chemotherapy, and (iv) < 18 years old.

According to the recruiting time, the study participants at Center 1 were divided into the development and internal validation cohorts at a ratio of 2:1. Furthermore, an external cohort from Center 2 was validated.

### CE lymphatic US examination and identification of sentinel LN

All US examinations were performed using a high-frequency, 5–18-MHz linear array transducer on the Acuson S2000 (Siemens Medical Systems, Erlangen, Germany), Logiq E9 (GE Health Care, Milwaukee, WI, USA), iU22, and EPIQ 7 (Philips-Advanced Technology Laboratories, Bothell, WA, USA) machines. The contrast agents SonoVue (Bracco Imaging, Milan, Italy) or Sonazoid (GE Healthcare AS, Oslo, Norway) were used for patients at a ratio of 1:1 according to the enrollment time. The contrast agent was mixed with 2 mL sterile saline. Approximately 0.5 mL of contrast agent was injected intradermally into the periareolar position of the affected breast. Up to 3 additional injections could be performed if the lymphatic channel or SLN was not clearly detected. The dual display mode showing both CE and grayscale US images was used to confirm the SLN.

All US physicians were trained in standard SLN CE lymphatic US. The CE lymphatic US examinations were performed by two US physicians (Z.Q.L. and C.L.G.) before the surgery on the same day. At the same time, the images were analyzed by the above two physicians on site. All US examination results, saved in cine video format, were independently reviewed by two other experienced US physicians (N.Z.H. and H.Y.X.) who were blinded to the pathological results of ALN status. Additional details regarding the procedure are given in Supplementary Material.

### Surgical management of ALNs

During the operation, methylene blue dye and indocyanine green were intradermally injected into the periareolar tissue to identify SLNs. LNs stained blue and/or green were considered SLNs. The surgeon determined whether the location of the SLN matched the location marked on the skin after CE lymphatic US. The microscopic inspection results of SLN biopsy or ALN dissection were regarded as the gold standard. Micrometastasis was defined as tumor deposits in the LN measuring > 0.2 mm but not exceeding 2 mm. Macrometastasis was defined as a deposit measuring ≥ 2 mm. Both micro- and macrometastases were defined as metastasis [19].

### Image analysis

**Fig. 1:**
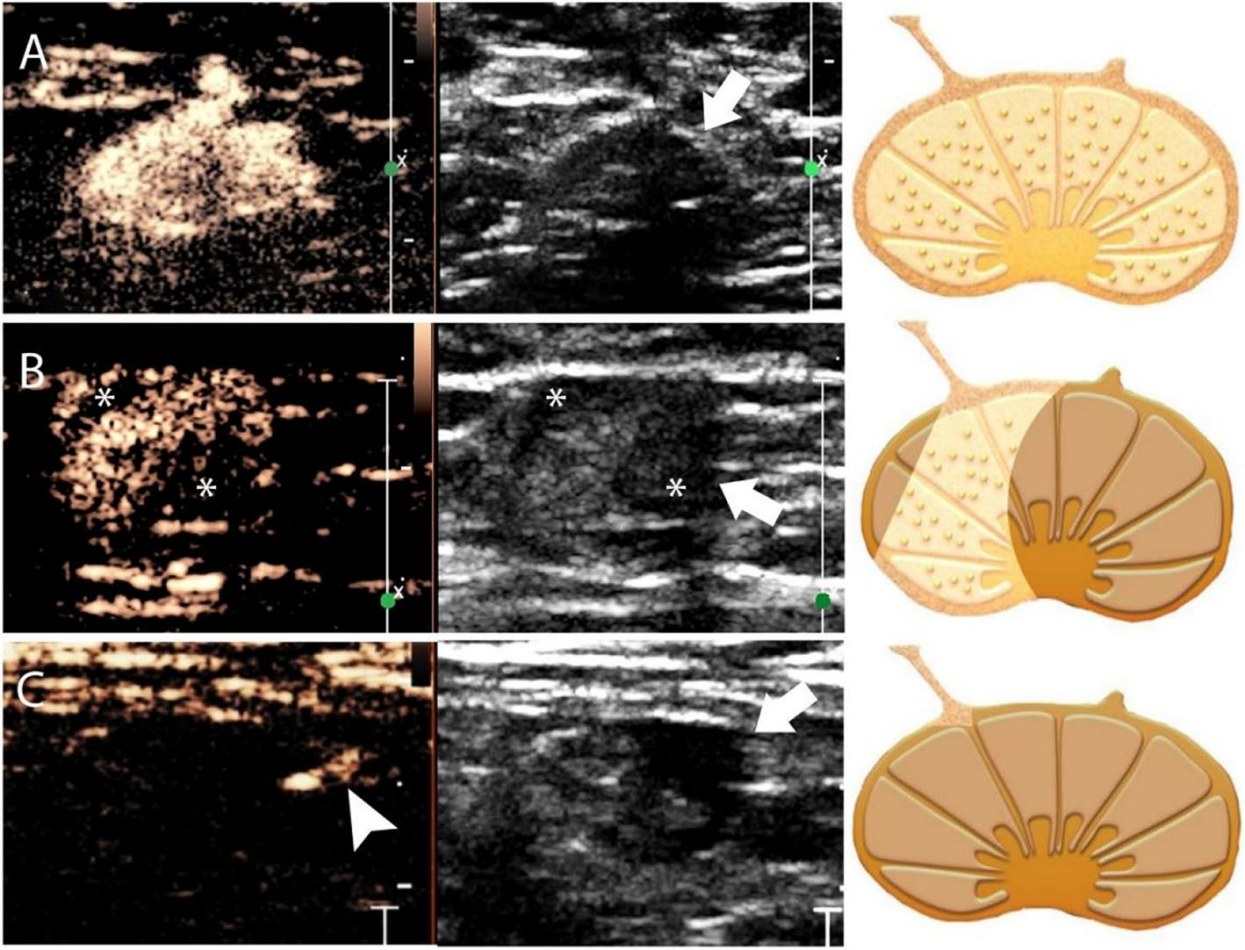
Enhancement patterns of the SLNs on contrast-enhanced lymphatic US. From left to right: the enhanced US image; the grayscale US image at the dual display; and the schematic illustration of enhanced and grayscale US images. **A** Pattern I, homogeneous enhancement. The echogenic contrast agent is evenly distributed within the SLN with a thin hypoechoic cortex (arrows) on the grayscale US. **B** Pattern II, inhomogeneous enhancement. The uneven distribution of the contrast agent with two filling defect areas (*) of the SLN is seen correlating to the focal eccentric cortical thickening (arrows) on grayscale US. **C** Pattern III, no enhancement. There is an enhanced lymphatic vessel (arrowhead), but no contrast agent accumulation is visible inside the SLN. The SLN shows focal eccentric cortical thickening on grayscale US (arrows). *SLN* sentinel lymph node

The enhancement patterns of the SLNs were classified into pattern I “homogeneous” when the entire lymph node showed bright with homogeneous enhancement (Supplementary Movie 1), pattern II “inhomogeneous” when the lymph node showed a focal non enhancement area (Supplementary Movie 2), "no enhancement" if the lymph node did not show enhancement and any contrast entering the node after the contrast injection under enhanced afferent lymphatic vessel guidance (Supplementary Movie 3) (Fig. 1) [13–17]. Each LN was classified as one of these patterns exclusively.

**Fig. 2:**
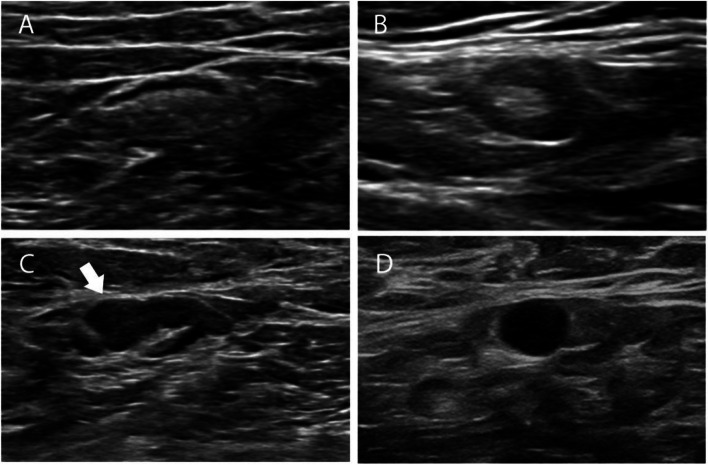
Cortical morphology of the SLNs on grayscale US. **A** Type I, SLN with thin hypoechoic cortex. The cortical thickness was less than 2.5 mm. **B** Type II, SLN with diffuse cortical thickening. The hypoechoic cortex is uniformly thicker than 3 mm. **C** Type III, SLN with focal eccentric cortical thickening (arrows). The cortex is thicker than 3 mm and the hilum is displaced. **D** Type IV, a round hypoechoic SLN with absent hilum. *SLN* sentinel lymph node

The cortical morphology of SLNs was classified into: type I, a thin hypoechoic cortex with a cortical thickness < 3 mm; type II, diffuse thickening of the hypoechoic cortex with a cortical thickness ≥ 3 mm; type III, focal eccentric thickening of the hypoechoic cortex with hilar displacement; or type IV, a round hypoechoic node with no hilum (Fig. 2) [20–23]. Each LN was classified as one of these types. For all disagreements between the two readers at Center 1, a consensus reading was then achieved (still blinded to the final ALN status). Intraobserver agreement was assessed by the physician (Z.Q.L.) re-evaluating nodal enhancement pattern and cortical morphology after a six-month interval.

### Construction and validation of the nomogram

The univariable and multivariate logistic regression was performed on the clinical, pathological, and US variables, as detailed in Supplementary Material. The performance of the nomogram was compared with that of the grayscale US and the lymphatic US with a predefined threshold. The optimal thresholds for lymphatic and grayscale US were determined using the sensitivity and specificity pair that yielded the highest Youden index.

### Statistical analysis

Continuous variables are expressed as the mean ± standard deviation (SD) or the median with full or interquartile range, and categorical variables are expressed as numbers and percentages (%). The difference between the two evaluations was compared using the Kruskal-Wallis H test for continuous data and the chi-squared test for categorical data. A calibration curve was used to assess the model fit in both the development and validation cohorts. The calibration slope, average errors (E average [Eaver]), maximal errors (E maximal [Emax]), and Brier score between the predicted and observed risk obtained from the calibration curve were evaluated [24]. The clinical utility of the model was evaluated with decision curve analysis [25]. Receiver operating characteristic (ROC) curve analysis and the corresponding AUC values were used to assess the discriminability of the diagnostic models. The AUCs were compared using the Delong method. The inter- and intra-reader agreements were assessed by using kappa statistics [26]. *p* < 0.05 was considered statistically significant. All statistical analyses were performed using R (http://www.R-project.org), EmpowerStats software (X&Y Solutions), and MedCalc (version 17.9.7).

## Results

### Patient characteristics of the development and validation cohorts

**Fig. 3:**
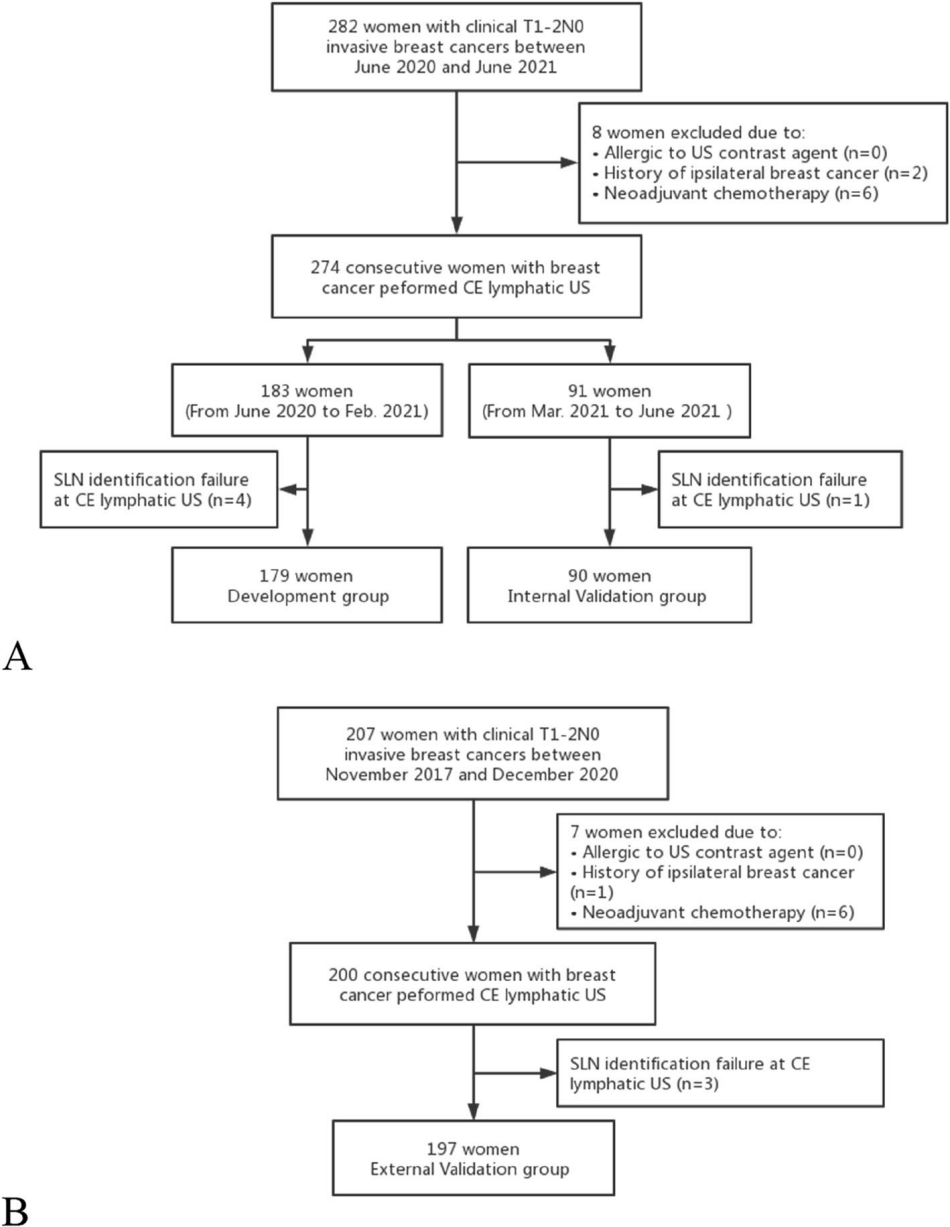
Flowchart of (**A**) development cohort, internal validation cohort and (**B**) external validation cohort in this study. *SLN* sentinel lymph node; *CE* contrast-enhanced

A total of 282 women with T1-2 invasive breast cancers and negative clinical palpable ALN were considered for inclusion and underwent lymphatic US examination between June 2020 and June 2021 at Center 1. Eight women were excluded owing to a history of ipsilateral breast cancer with axillary surgery or radiotherapy (*n* = 2), and neoadjuvant chemotherapy (*n* = 6). Five women were excluded due to failure of identifying the SLNs on the lymphatic US. The identification rate of SLNs by the CE lymphatic US was 98.2% (269/274), and the identification rate of Sonovue and Sonazoid was 97.7% (172/176) and 99.0% (97/98), respectively. Ultimately, 179 women between June 2020 and February 2021 formed the development cohort, and 90 women between March 2021 and June 2021 formed the internal validation cohort (Table 1) (Fig. 3).

**Table 1:** Characteristics of the development and validation cohorts

Characteristic	(*n* = 466)	(*n* = 179)	(*n* = 90)	(*n* = 197)	
	Total	Development cohort	Internal validation cohort	External validation cohort	*p* value
Age (y)^a^	52.8 ± 11.7	50.3 ± 10.8	54.6 ± 11.3	54.4 ± 12.2	.001
Clinical T stage					
T1	242 (51.9%)	89 (49.7%)	48 (53.3%)	105 (53.3%)	.75
T2	224 (48.1%)	90 (50.3%)	42 (46.7%)	92 (46.7%)	
Ultrasound tumor size (cm)^b^	2.1 ± 0.9	2.0 (1.4–2.6)	1.9 (1.4–2.3)	1.9 (1.4–2.5)	.45
Histologic type					.81
Invasive ductal carcinoma	378 (81.1%)	143 (79.9%)	71 (78.9%)	164 (83.2%)	
Invasive lobular carcinoma	21 (4.5%)	10 (5.6%)	4 (4.4%)	7 (3.6%)	
Other types	67 (14.4%)	26 (14.5%)	15 (16.7%)	26 (13.2%)	
Histologic grade					.01
Low	63 (13.5%)	27 (15.1%)	15 (16.7%)	21 (10.7%)	
Intermediate	260 (55.8%)	107 (59.8%)	58 (64.4%)	95 (48.2%)	
High	126 (27.0%)	41 (22.9%)	17 (18.9%)	68 (34.5%)	
Not applicable	17 (3.6)	4 (2.2%)	0 (0.0%)	13 (6.6%)	
Lymphovascular invasion					< .001
No	363 (80.3%)	153 (85.5%)	79 (87.8%)	142 (72.1%)	
Yes	92 (19.7%)	26 (14.5%)	11 (12.2%)	55 (27.9%)	
ER					.001
Negative	100 (21.5%)	30 (16.8%)	12 (13.3%)	58 (29.4%)	
Positive	366 (78.5%)	149 (83.2%)	78 (86.7%)	139 (70.6%)	
PR					.01
Negative	145 (31.1%)	46 (25.7%)	23 (25.6%)	76 (38.6%)	
Positive	321 (68.9%)	133 (74.3%)	67 (74.4%)	121 (61.4%)	
Not applicable					
HER2					.04
Negative	374 (80.3%)	148 (82.7%)	78 (86.7%)	148 (75.1%)	
Positive	92 (19.7%)	31 (17.3%)	12 (13.3%)	49 (24.9%)	
Type of breast surgery					< .001
Breast conserving surgery	181 (38.8%)	86 (48.0%)	53 (58.9%)	42 (21.3%)	
Mastectomy	285 (61.2%)	93 (52.0%)	37 (41.1%)	155 (78.7%)	
Contrast agent					
Sonovue	254 (54.5%)	150 (83.8%)	0 (0%)	104 (52.8%)	< .001
Sonazoid	212 (45.5%)	29 (16.2%)	90 (100%)	93 (47.2%)	
Mean number of SLNs by blue or green dyes^c^	3.0 (0.0–12.0)	4 (0–12)	4 (1–11)	2 (0–6)	< .001
Mean number of SLNs by CE lymphatic US^c^	1 (1–5)	1 (1–5)	1 (1–4)	1 (1–4)	< .001
SLN biopsy results					*.*54
No metastasis	326 (70.0%)	119 (66.5%)	65 (72.2%)	142 (72.1%)	
Micrometastasis	11 (2.4%)	3 (1.7%)	2 (2.2%)	6 (3.0%)	
Macrometastasis	129 (27.7%)	57 (31.8%)	23 (25.6%)	49 (24.9%)	
Type of axillary surgery					.36
SLN biopsy	350 (75.1%)	129 (72.1%)	72 (80.0%)	149 (75.6%)	
ALN dissection	116 (24.9%)	50 (27.9%)	18 (20.0%)	48 (24.4%)	
Number of metastatic ALN					.91
0	326 (70.0%)	119 (66.5%)	65 (72.2%)	142 (72.1%)	
1	56 (12.0%)	25 (14.0%)	10 (11.1%)	21 (10.7%)	
2	33 (7.1%)	14 (7.8%)	5 (5.6%)	14 (7.1%)	
≥ 3	51 (10.9%)	21 (11.7%)	10 (11.1%)	20 (10.2%)	

Unless otherwise specified, data are numbers of patients, with percentages in parentheses

*ER* estrogen receptor, *PR* progesterone receptor, *HER2* human epidermal growth factor receptor 2, *SLN* sentinel lymph node, *ALN* axillary lymph node

^a^Data are means ± standard deviations

^b^Data are medians, with interquartile range in parentheses

^c^Data are medians, with ranges in parentheses

A total of 207 women at Center 2 were recruited between November 2017 and December 2020. Seven women were excluded owing to a history of ipsilateral breast cancer (*n* = 1) or neoadjuvant chemotherapy (*n* = 6). The identification rate of SLNs by the CE lymphatic US was 98.5% (197/200), and the identification rate of Sonovue and Sonazoid was 99.0% (104/105) and 97.9% (93/95), respectively. Finally, 197 women formed the external validation cohort (Fig. 3). No patient had an ultrasound contrast reaction.

The cohorts were comparable in terms of clinical T stage (*p* = 0.75), tumor histologic type (*p* = 0.81), tumor size (*p* = 0.45), axillary surgery type (*p* = 0.36), SLN biopsy results (*p* = 0.54), and ALN pathologic status (*p* = 0.91) (Table 1). However, the three cohorts differed in terms of age (*p* = 0.001), histologic grade (*p* = 0.01), lymphovascular invasion (*p* < 0.001), estrogen receptor (*p* = 0.001), progesterone receptor (*p* = 0.01), human epidermal growth factor receptor 2 (*p* = 0.04), breast surgery type (*p* < 0.001), contrast agent (*p* < 0.001), mean number of SLNs identified by CE lymphatic US (*p* < 0.001), and blue dye and ICG (*p* < 0.001) (Table 1).

### US findings according to pathologic ALN status

The US findings of the SLNs according to final ALN status are shown in Table 2. A total of 53.3% (8/15), 50.0% (4/8), and 63.6% (7/11) of SLNs with no enhancement patterns were ≥ 3 metastatic ALNs in the development, internal and external validation cohorts. SLNs with an absent hilum found in 66.7% (4/6), 100% (2/2), and 54.5% (6/11) of patients in the three cohorts were ≥ 3 metastatic ALNs. Most of the SLNs with homogeneous patterns, thin cortex, and diffuse cortical thickening were 0–2 metastatic ALNs.

**Table 2:** US findings of sentinel lymph nodes according to pathologic node status

	Development cohort				Internal validation cohort				External validation cohort			
Characteristic	Total (*n* = 179)	Metastatic ALNs			Total (*n* = 90)	Metastatic ALNs			Total (*n* = 197)	Metastatic ALNs		
		0 (*n* = 119)	1–2 (*n* = 39)	≥ 3 (*n* = 21)		0 (*n* = 65)	1–2 (*n* = 15)	≥ 3 (*n* = 10)		0 (*n* = 142)	1–2 (*n* = 35)	≥ 3 (*n* = 20)
Enhancement patterns												
Homogeneous enhancement	42	40 (95.2%)	2 (4.8%)	0 (0.0%)	17	15 (88.2%)	2 (11.8%)	0 (0.0%)	133	118 (88.7%)	13 (9.8%)	2 (1.5%)
Inhomogeneous enhancement	122	75 (61.5%)	34 (27.9%)	13 (10.6%)	65	48 (73.8%)	11 (16.9%)	6 (9.2%)	53	22 (41.5%)	20 (37.7%)	11 (20.8%)
No enhancement	15	4 (26.7%)	3 (20.0%)	8 (53.3%)	8	2 (25.0%)	2 (25.0%)	4 (50.0%)	11	2 (18.2%)	2 (18.2%)	7 (63.6%)
Cortical morphology												
Thin cortex	88	72 (81.8%)	15 (17.0%)	1 (1.1%)	57	49 (86.0%)	7 (12.3%)	1 (1.7%)	138	122 (88.4%)	12 (8.7%)	4 (2.9%)
Diffuse cortical thickening	39	27 (69.2%)	6 (15.4%)	6 (15.4%)	14	12 (85.7%)	2 (14.3%)	0 (0.0%)	15	11 (73.3%)	2 (13.3%)	2 (13.3%)
Focal eccentric cortical thickening	46	20 (43.5%)	16 (34.8%)	10 (21.7%)	17	4 (23.5%)	6 (35.3%)	7 (41.2%)	33	8 (24.2%)	17 (51.5%)	8 (24.2%)
Absent hilum	6	0 (0.0%)	2 (33.3%)	4 (66.7%)	2	0 (0.0%)	0 (0.0%)	2 (100.0%)	11	1 (9.1%)	4 (36.4%)	6 (54.5%)

Data are numbers of patients, and the data in parentheses are percentages

*ALN* axillary lymph node

### Factors associated with three or more metastatic ALNs

In the development cohort of 179 women, among the clinicopathologic and US variables, the enhancement patterns and cortical morphology of SLNs were associated with the final ALN disease burden in the univariable analysis. However, age, tumor size, quadrant, receptor status, histologic type, histologic grade, and lymphovascular invasion were not associated with having ≥ 3 metastatic ALNs. In the multivariate analysis, SLNs with no enhancement (odds ratio (OR), 15.3; 95% CI: 3.4, 68.1; *p* = 0.01), diffuse cortical thickening (OR, 19.1; 95% CI: 2.0, 182.9; *p* = 0.01), focal eccentric cortical thickening (OR, 27.7; 95% CI: 3.1, 248.1;* p* = 0.003), and an absent hilum (OR, 169.7; 95% CI: 10.4, 2755.8; *p* < 0.001) were independently associated with the presence of ≥ 3 metastatic ALNs (Table 3).

**Table 3:** Univariable and multivariate logistic regression analyses of factors associated with ≥ 3 metastatic nodes in the development cohort

Variables	Univariable analysis		Multivariate analysis	
	OR (95% CI)	*p*	OR (95% CI)	*p*
Age	1.0 (0.9, 1.0)	.33		
Clinical T stage				
T1	1.0			
T2	0.9 (0.4, 2.2)	.80		
ER-positive	4.5 (0.6, 34.9)	.15		
PR-positive	3.7 (0.8, 16.4)	.09		
HER2-positive	0.8 (0.2, 2.8)	.70		
Histologic type				
Ductal	1.0			
Lobular	1.7 (0.3, 8.8)	.51		
Other	0.3 (0.0, 2.2)	.22		
Histologic grade				
Low	1.0			
Intermediate	1.9 (0.4, 8.8)	.42		
High	1.7 (0.3, 9.7)	.53		
Lymphovascular invasion	1.5 (0.4, 4.7)	.53		
US enhancement patterns				
Homogeneous/inhomogeneous enhancement	1.0	< .001	1.0	
No enhancement	13.3 (4.2, 42.4)	< .001	15.3 (3.4, 68.1)	.01
US cortical morphology				
Thin cortex	1.0			
Diffuse cortical thickening	15.8 (1.8, 136.4)	.01	19.1 (2.0, 182.9)	.01
Focal eccentric cortical thickening	24.2 (3.0, 195.8)	.003	27.7 (3.1, 248.1)	.003
Absent hilum	174.0 (12.9, 2345.8)	< .001	169.7 (10.4, 2755.8)	< .001

Data in parentheses are 95% confidence intervals

*ER* estrogen receptor, *PR* progesterone receptor, *HER2* human epidermal growth factor receptor 2

### Nomogram predicting three or more metastatic ALNs

**Fig. 4:**
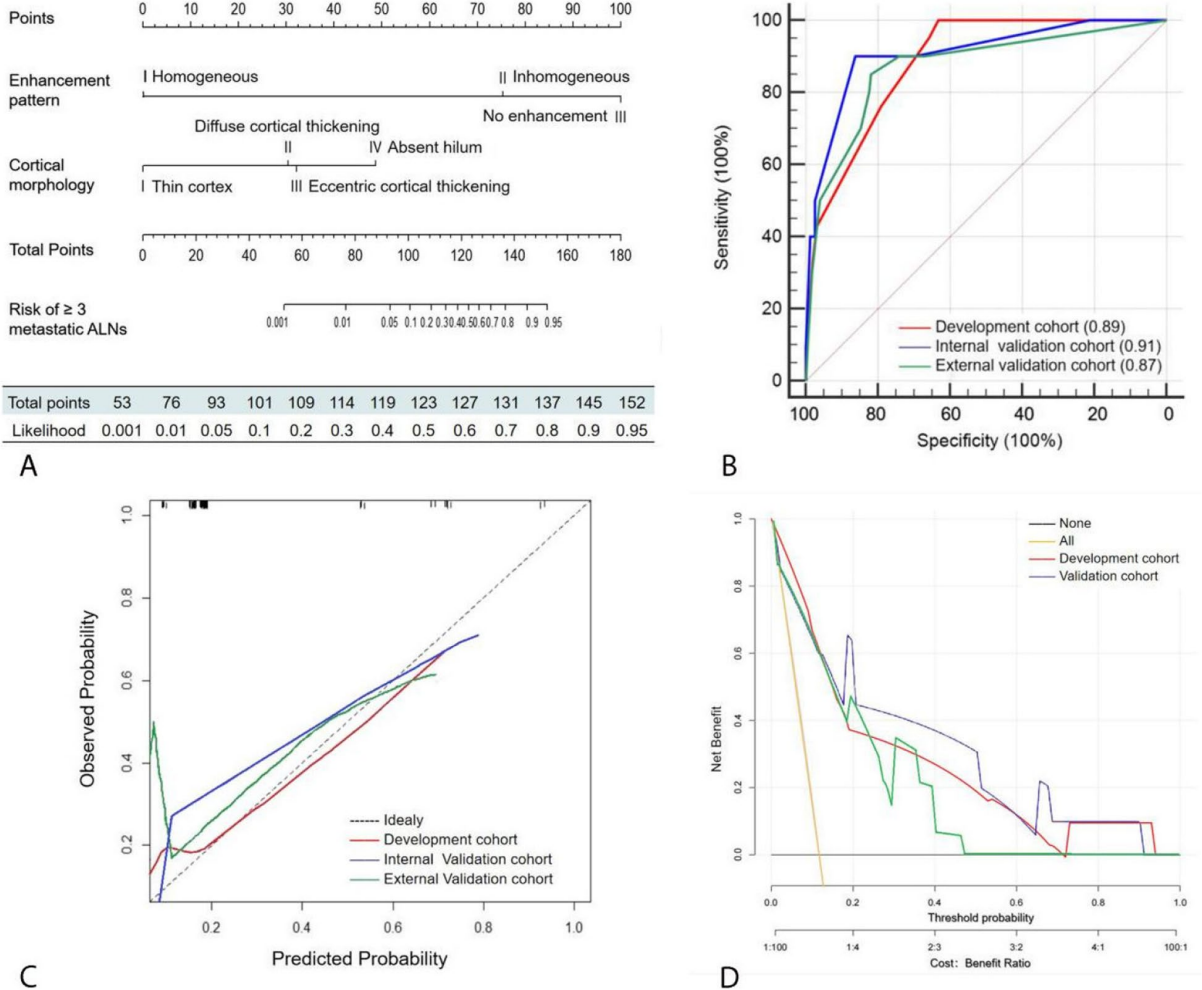
Development and validation of a nomogram to predict ≥ 3 metastatic ALNs. **A** Nomogram combining contrast-enhanced lymphatic US and grayscale US findings. Value assigned to each factor was scored on a scale of 0 to 100. By adding scores for each factor, one can obtain a total score. On the basis of the total score, the probability of having ≥ 3 metastatic ALNs is displayed by projecting the score to the bottom risk axis. **B** The receiver operating characteristic curve was obtained in the development (red line), the internal validation (blue line), and the external validation (green line) cohorts. **C** Calibration plot obtained in the development (red line), the internal validation (blue line), and the external validation (green line) cohorts. In calibration plots, a dotted line at a 45° angle represents perfect calibration. The nomogram-predicted probability of having ≥ 3 metastatic ALNs is plotted on the x-axis; the actual probability of having ≥ 3 metastatic ALNs is plotted on the *y*-axis. **D** Decision curves for having ≥ 3 metastatic ALNs in the development (red line), the internal validation (blue line), and the external validation (green line) cohorts. The yellow and black lines (horizontal) represent the scenarios where all or none of the women would be prospectively determined by the risk model, respectively. The decision lines demonstrate the net benefit of the risk model dependent at the chosen risk threshold for having ≥ 3 metastatic ALNs. *ALN* axillary lymph node

The variables, including clinical characteristics, pathological types, CE lymphatic US enhancement patterns, and grayscale US findings of SLNs were used to build the nomogram (Fig. 4A). The ROC curves showed good discrimination, with an AUC of 0.89 (95% CI: 0.83, 0.93) in the development cohort, 0.91 (95% CI: 0.83, 0.96) in the internal validation cohort, and 0.87 (95% CI: 0.78, 0.96) in the external validation cohort (Fig. 4B).

The calibration plots showed good estimation in predicting ≥ 3 metastatic ALNs. There was no significant difference between the predicted and observed probabilities in the development (slope = 1.0, *p* = 0.82, Eaver = 0.014, Emax = 0.104, Brier = 0.071), internal validation (slope = 1.0, *p* = 0.81, Eaver = 0.044, Emax = 0.160, Brier = 0.066), or external validation cohorts (slope = 1.0, *p* = 0.80, Eaver = 0.043, Emax = 0.422, Brier = 0.067) (Fig. 4C). Decision curve analysis demonstrated that the nomogram provided benefit across the range of reasonable threshold probabilities (Fig. 4D).

**Fig. 5:**
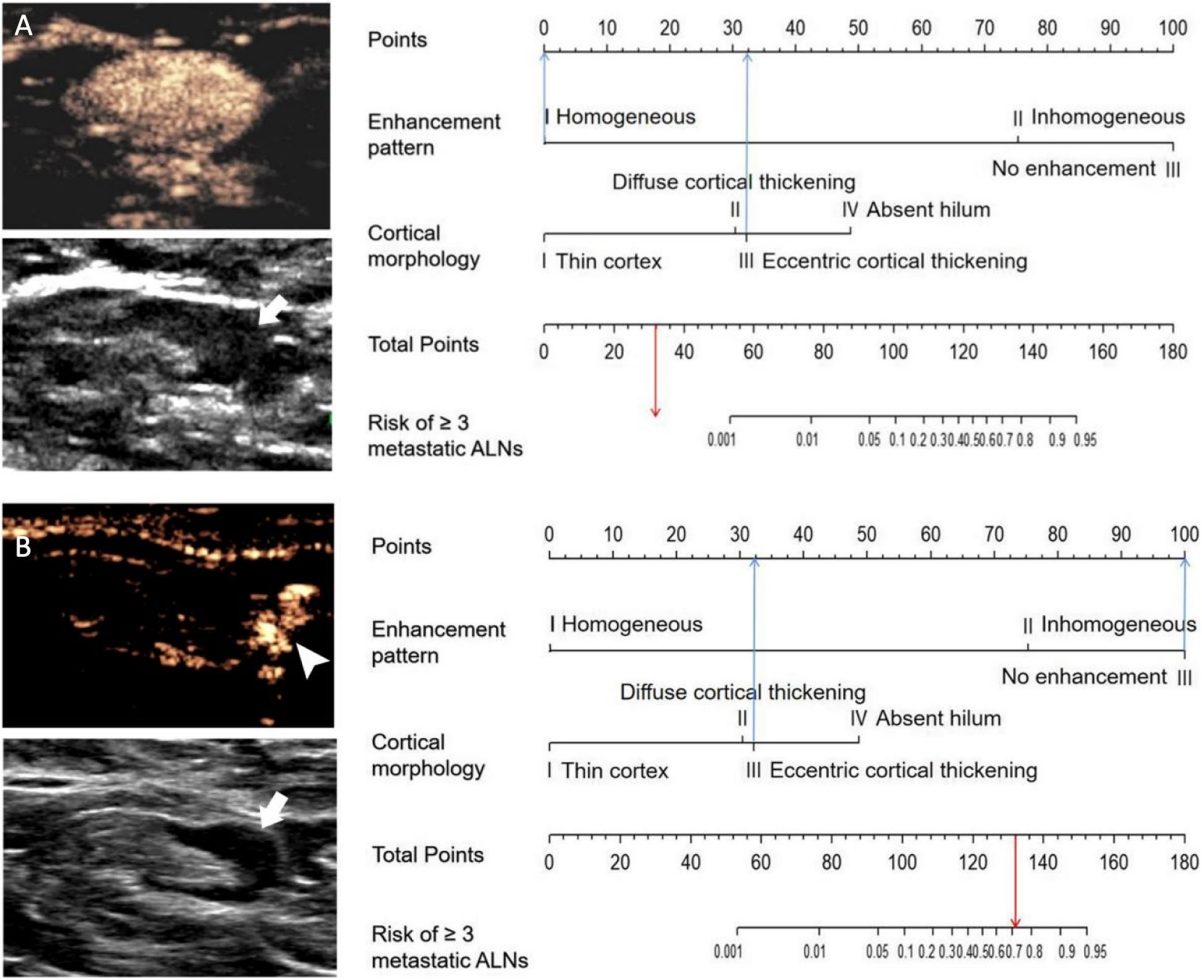
Examples of the nomogram in clinical practice. Figures illustrate the process of calculating the risk scores in low probability (**A**) and high probability (**B**) of having ≥ 3 metastatic SLNs using the nomogram. **A** The US findings of SLN in a 57-year-old woman with clinical T1 invasive carcinoma and no ALN metastasis. The SLN showed a homogeneous enhancement pattern and the cortical morphology of eccentric thickening (arrows). The total score is 32, which corresponds to a < 0.1% probability of having ≥ 3 metastatic ALNs. **B** The US findings of SLN in a 61-year-old woman with clinical T1 invasive carcinoma and 3 metastatic ALNs. The SLN showed no enhancement pattern and the cortical morphology of eccentric thickening (arrows). An arrowhead indicates the enhanced lymphatic vessels. The total score is 132, which corresponds to a 71.7 % probability of having ≥ 3 metastatic ALNs. *ALN* axillary lymph node, *SLN* sentinel lymph node

A probability of 40% was chosen as the threshold for increasing the positive predicted value without sacrificing sensitivity (Table 4). In the internal validation cohort, of the 84 women with a nomogram-based calculated probability of less than 40%, 78 indeed had two or fewer metastatic ALNs, resulting in a negative predictive value of 92.9% (78/84). Of the 6 women identified as “positive” (i.e., those with a nomogram-based probability greater than 40%), 4 had ≥ 3 metastatic ALNs, resulting in a positive predictive value of 66.7% (4/6). In the external cohort, the negative and positive predictive values were 92.6% (174/188) and 66.7% (6/9), respectively. The examples of the clinical use of the nomogram are shown in Fig. 5. Two examples of false-positive cases are shown in Supplementary Fig. 2.

**Table 4:** The performance of nomograms for the prediction of ≥ 3 metastatic lymph nodes

Cohort	Threshold	Sensitivity (%)	Specificity (%)	Accuracy (%)	PPV (%)	NPV (%)	AUC
Development cohort (*n* = 179)	30%	42.9 (9/21) [21.8, 66.0]	96.8 (153/158) [92.8, 99.0]	90.5 (162/179) [85.2, 94.4]	64.3 (9/14) [35.1, 87.2]	92.7 (153/165) [87.6, 96.2]	0.89 [0.83, 0.93]
	40%	33.3 (7/21) [14.6, 57.0]	98.1 (155/158) [94.6, 99.6]	90.5 (162/179) [85.2, 94.4]	70.0 (7/10) [34.8, 93.3]	91.7 (155/169) [86.5, 95.4]	
	50%	23.8 (5/21) [8.2, 47.2]	98.7 (156/158) [95.5, 99.9]	89.9 (161/179) [84.6, 93.4]	71.3 (5/7) [29.0, 96.3]	90.7 (156/172) [85.3, 94.6]	
Internal validation cohort (*n* = 90)	40%	40.0 (4/10) [12.2, 73.8]	97.5 (78/80) [91.3, 99.7]	91.11 (82/90) [83.23, 96.1]	66.7 (4/6) [22.3, 95.7]	92.9 (78/84) [85.1, 97.3]	0.91 [0.83, 0.96]
External validation cohort (*n* = 197)	40%	30.0 (6/20) [11.9, 54.3]	98.3 (174/177) [95.1, 99.7]	91.4 (180/197) [86.5, 94.9]	66.7 (6/9) [29.9, 92.5]	92.6 (174/188) [87.8, 95.9 ]	0.87 [0.78, 0.96]

Data in parentheses are numbers of patients used to calculate percentages. Data in brackets are 95% confidence intervals. Other data are reported as percentages

*PPV* positive predictive value, *NPV* negative predictive value, *AUC* area under the receiver operating characteristic curve

This resulted in a sensitivity and specificity of 23.5% and 98.3% for grayscale US alone, 37.3% and 96.4% for lymphatic US alone, and 33.3% and 98.1% for the nomogram to predicting ≥ 3 metastatic ALNs, respectively. The lymphatic US showed the highest sensitivity while the grayscale US showed the highest specificity. The nomogram possessed the two single methods advantages and showed the highest AUC of 0.88. The nomogram also achieved the highest accuracy of 91.0% (95% CI: 88.0, 93.4) and positive predictive values of 68% (95% CI: 46.5, 85.1) at predicting ≥ 3 metastatic ALNs (Supplementary Table 1). Additionally, the diagnostic value of two different contrast agents was compared. The AUC was not significantly different between the Sonovue (0.88 (0.83, 0.92)) and Sonazoid (0.88 (0.83, 0.92)) (*p* = 0.940) for predicting ≥ 3 metastatic ALNs (Supplementary Table 2).

### Inter- and intraobserver agreement for US findings

The diagnostic performance of the nomogram according to two readers from Center 1 is detailed in Supplementary Fig. 3. We found no evidence of a difference between readers 1 and 2 or in the consensus reading of the nomogram. The two readers identically classified the enhancement patterns in 91.1% (245/269) and the cortical morphology in 84.0% (226/269) of SLNs (Supplementary Table 3).

The inter-observer agreement was strong for the enhancement patterns (*κ* = 0.81 in the development cohort and *κ* = 0.88 in the validation cohort) and moderate for cortical morphology (*κ* = 0.78 in the development cohort and *κ* = 0.73 in the validation cohort). The intra-observer agreement was strong (*κ* = 0.80–0.84 for enhancement patterns and *κ* = 0.89–0.90 for cortical morphology)

## Discussion

In the post-Z0011 era, there is an increasing demand for the development of a more reliable imaging method that can accurately predict ≥ 3 metastatic ALNs in clinical T1-2N0 breast cancer. In this study, we constructed a nomogram combining lymphatic and grayscale US findings of SLNs after multivariate analysis to predict ≥ 3 metastatic ALNs. The nomogram showed good discriminability (AUC, 0.91 and 0.87) and calibration (calibration slope, 1.0) in the validation cohorts. When using a threshold of 40%, the nomogram identified patients with ≥ 3 metastatic ALNs with a specificity of 97.5–98.3% and a positive predictive value of 66.7% in the validation cohorts. The nomogram based on conventional US and CE lymphatic US was demonstrated to be feasible for the ALN staging.

The nomogram based on the CE lymphatic US and grayscale US is more applicable to the post-Z0011 era and has undeniably shaken up the management of the axilla in early breast cancer, which can be utilized to triage a patient to receive the neoadjuvant systemic treatment or ALN dissection directly. By setting the threshold at 40%, we achieved the highest positive predicted value thus minimizing over-treatment of patients with low metastatic burden. The low sensitivity in the development cohort (33.3%) resulted in patients with high tumor burden that could be missed by the nomogram. One might expect such patients would receive the standard SLN biopsy and could not be missed.

SLNs with inhomogeneous or nonenhancement on the CE lymphatic US can occur in both low and high-metastatic burden ALNs with an overlap, causing false positive cases for predicting ≥ 3 metastatic ALN. Previous studies have reported that some special enhancement patterns, such as cortical filling and complete annular high enhancement, even in inhomogeneous patterns, also suggest a benign SLN [27, 28]. Further classifying inhomogeneous patterns could help to reduce the false-positive rate. The enhancement pattern is also affected by regional tissue and lymphatic reflux pressure [29]. There is a limitation of the naked eye in observing SLN enhancement patterns, which also can cause false-positive results on the benign SLNs. Some studies have demonstrated that radiomics data based on grayscale images of from primary tumor US have good performance in predicting ALN metastasis [30, 31]. Applying radiomics to CE lymphatic images of SLN can rapidly extract quantitative features and accurately identify contrast-enhanced patterns, which might improve diagnostic efficiency. Further studies are expected to explore the value of radiomics and artificial intelligence for SLN status diagnosis.

Two different commercially available contrast agents were used to identify the SLNs in this study. Compared with Sonovue, Sonazoid exhibits an additional Kupffer phase and has good stability [32]. In our study, both Sonovue and Sonazoid have great potential for locating SLNs. No significant differences in the number of detected SLNs and the diagnosis of metastatic ALNs were observed, which is the same as a previous study with 205 women with clinical T1–2N0 breast cancer [33].

In addition to the identification of suspicious nodes by imaging, axillary metastases are also related to clinicopathologic characteristics such as patient age, tumor size, type, histologic grade, and lymphovascular invasion [18, 34]. However, in our study, only US image findings were associated with ≥ 3 metastatic ALNs, which is consistent with the result of some previous studies [7, 35]. Although there were several differences in the clinical and histopathological variables between the development and validation groups, our nomogram showed similar specificity and accuracy in the two cohorts and appears to be a robust tool to provide preoperative information.

Our study had some limitations. First, only one SLN identified by CE lymphatic US was included in the analysis. For some patients, more than one SLN was enhanced, evaluating all enhanced SLNs would help improve the false-positive results. Second, the previous biopsy procedure might affect the display of lymphatic vessels. Third, the CE lymphatic US-enhanced image reading is operator-dependent, and a learning curve is needed.

## Conclusions

A nomogram combining CE lymphatic US and grayscale US findings of SLNs could identify early breast cancer patients with low or high axillary tumor burden preoperatively, which is more applicable to the Z0011 era. As the number of institutions using lymphatic US for SLN identification continues to increase, our nomogram could be useful in aiding multidisciplinary treatment decision-making for patients with early breast cancer.

### Supplementary Information

**Additional file 1:Supplementary Table 1.** Comparison of contrast-enhanced Lymphatic US, Grayscale US, and Nomogram for predicting ≥ 3 Metastatic ALNs. **Supplementary Table 2.** Comparison of Contrast agent for predicting ≥ 3 Metastatic ALNs. **Supplementary Table 3.** Analysis of US findings of SLN according to reader 1, reader 2 at Center 1, and consensus reading. **Supplementary Figure 1.** Images in a 30-year-old woman with invasive ductal carcinoma in the left breast. A. Image of contrast agent injected intradermally into the periareolar area of the affected breast. B. The CEUS image of SLN. The SLN (arrowhead) was visualized along the enhanced lymphatic channel (arrow) and showed inhomogeneous pattern. C. The lymphatic channel (arrow) and location of the SLN (arrowhead) was marked on the skin. CEUS = contrast-enhanced ultrasound, SLN = sentinel lymph node. **Supplementary Figure 2.** Two false positive examples of the nomogram in clinical practice. A. The lymphatic and grayscale US findings of SLN in a 53-year-old woman with clinical T2 invasive carcinoma and 2 SLNs metastasis. The SLN showed no enhancement pattern and the cortical morphology of concentric thickening (arrows). An arrowhead indicates the enhanced lymphatic vessels. The total score is 130, which corresponds to about a 67.5% probability of having ≥ 3 metastatic SLNs. B. The lymphatic and grayscale US findings of SLN in a 60-year-old woman with clinical T2 invasive carcinoma and 1 metastatic SLN. The SLN showed an inhomogeneous enhancement pattern and the absent hilum (arrows). An arrowhead indicates the enhanced lymphatic vessels. The total score is about 123, which corresponds to a 50 % probability of having ≥ 3 metastatic ALNs. ALN = axillary lymph node, SLN = sentinel lymph node. **Supplementary Figure 3.** Area under the curve (AUC) for predicting ALN disease burden of ≥ 3 metastatic ALNs according to reader 1, reader 2 and consensus reading at Center 1. A. AUC in development cohort. AUC of was 0.85 (95% CI: 0.79, 0.90), 0.87 (95% CI: 0.82, 0.92) and 0.89 (95% CI: 0.83, 0.93) for reader 1, reader 2 and consensus reading respectively. No statistically significant difference was found between the two readers (DeLong test, *p* = 0.246). B. AUC in validation cohort. AUC was 0.90 (CI 95%: 0.82, 0.96), 0.92 (95% CI: 0.84, 0.97) and 0.91 (95% CI: 0.82, 0.96) for reader 1, reader 2 and consensus reading respectively. No statistically significant difference were found between the two readers (DeLong test, *p* = 0.235). AUC = Area under the curve, ALN = axillary lymph node.

**Additional file 2.**

** Additional file 3.**

**Additional file 4.**

## Data Availability

The datasets generated or analyzed during the study are available from the corresponding author on reasonable request.

## Abbreviations

ALN: Axillary lymph node
AUC: Area under the receiver operating characteristic curve
CE: Contrast-enhanced
OR: Odds ratio
ROC: Receiver operating characteristic
SLN: Sentinel lymph nodes
US: Ultrasound
