# Supplementary material for: Predicting three or more metastatic nodes using contrast-enhanced lymphatic US findings in early breast cancer
Source: Insights Imaging. 2024 Mar 25;15:86. doi: 10.1186/s13244-024-01648-1 (PMC10961298; doi:10.1186/s13244-024-01648-1)
Supplement: Supplementary file 1 — Additional file 1:Supplementary Table 1. Comparison of contrast-enhanced Lymphatic US, Grayscale US, and Nomogram for predicting ≥ 3 Metastatic ALNs. Supplementary Table 2. Comparison of Contrast agent for predicting ≥ 3 Metastatic ALNs. Supplementary Table 3. Analysis of US findings of SLN according to reader 1, reader 2 at Center 1, and consensus reading. Supplementary Figure 1. Images in a 30-year-old woman with invasive ductal carcinoma in the left breast. A. Image of contrast agent injected intradermally into the periareolar area of the affected breast. B. The CEUS image of SLN. The SLN (arrowhead) was visualized along the enhanced lymphatic channel (arrow) and showed inhomogeneous pattern. C. The lymphatic channel (arrow) and location of the SLN (arrowhead) was marked on the skin. CEUS = contrast-enhanced ultrasound, SLN = sentinel lymph node. Supplementary Figure 2. Two false positive examples of the nomogram in clinical practice. A. The lymphatic and grayscale US findings of SLN in a 53-year-old woman with clinical T2 invasive carcinoma and 2 SLNs metastasis. The SLN showed no enhancement pattern and the cortical morphology of concentric thickening (arrows). An arrowhead indicates the enhanced lymphatic vessels. The total score is 130, which corresponds to about a 67.5% probability of having ≥ 3 metastatic SLNs. B. The lymphatic and grayscale US findings of SLN in a 60-year-old woman with clinical T2 invasive carcinoma and 1 metastatic SLN. The SLN showed an inhomogeneous enhancement pattern and the absent hilum (arrows). An arrowhead indicates the enhanced lymphatic vessels. The total score is about 123, which corresponds to a 50 % probability of having ≥ 3 metastatic ALNs. ALN = axillary lymph node, SLN = sentinel lymph node. Supplementary Figure 3. Area under the curve (AUC) for predicting ALN disease burden of ≥ 3 metastatic ALNs according to reader 1, reader 2 and consensus reading at Center 1. A. AUC in development cohort. AUC of was 0.85 (95% CI: 0.79 [file 13244_2024_1648_MOESM1_ESM.pdf]

Predicting Three or More Metastatic Nodes Using Contrast-enhanced Lymphatic US Findings in Early Breast Cancer

ELECTRONIC SUPPLEMENTARY MATERIAL

CE lymphatic US Examination and Identification of Sentinel LN

We used SonoVue (Bracco Imaging, Milan, Italy) during the first half of the study, and changed the Sonazoid (GE Healthcare AS, Oslo, Norway) during the latter half of the study. The two contrast agents SonoVue or Sonazoid were used for patients at a ratio of 1:1 according to the enrollment time. The contrast agent (SonoVue, Bracco Imaging, Milan, Italy or Sonazoid, GE Healthcare AS, Oslo, Norway) was mixed with 2 mL sterile saline. Approximately 0.5 mL of contrast agent was injected intradermally into the periareolar position of the affected breast, and the injection area was massaged for 10-30 s. Up to 3 additional injections could be performed if the lymphatic channel or SLN was not clearly detected. With the mode of contrast pulse sequences (CPS), the low mechanical index (MI) value was set to 0.06-0.10. The average scan time was 10 minutes (range, 5-15 minutes). The locations of the enhanced lymphatic duct and SLNs were marked on the skin surface under the guidance of lymphatic US. This serves as a road map so that the SLNs can be identified easily by surgeons. The dual display mode showing both CE and grayscale US images was used to confirm the SLN.

All US physicians were trained in standard SLN CE lymphatic US (ZQL and NZH from Center 1 with 21 and 5 years of experience in breast US, 10 and 5 years in CE lymphatic US; CLG and HYX from Center 2 with 15 and 5 years in breast US, 10 and 3 years in CE lymphatic US). The CE lymphatic US examinations were performed by two US physicians (ZQL from Center 1; CLG from Center 2) before the surgery on the same day. At the same time, the images were analyzed by the above two physicians on site. All US examination results, saved in cine video format, were independently reviewed by two other experienced US physicians (NZH from Center 1; HYX from Center 2) who were blinded to the pathological results of ALN status. Additional details regarding the procedure are given in Supplementary Material.

The first enhanced LN was considered as SLN and used for analysis if only one SLN was detected. If more than one SLNs were enhanced, the SLN with the most suspicious enhancement pattern or the first enhanced SLN via the efferent lymphatic vessels was used for analysis.

Construction and Validation of the Nomogram

To build the diagnostic model, multicollinearity analysis was performed on the clinical, pathological and US variables. By calculating the variance inflation factor, the variables with a variance inflation factor value >10 were excluded. The univariable and multivariate logistic regression analyses were employed to screen the potential risk factors for having ≥3 metastatic ALNs, and the variables that correlated with a high risk for having ≥3 metastatic ALNs were entered into the multivariate logistic regression analysis and utilized to build the nomogram. The nomogram was validated in an independent cohort.

Supplementary Table 1. Comparison of contrast-enhanced Lymphatic US, Grayscale US, and Nomogram for predicting ≥ 3 Metastatic ALNs

Imaging Methods	Threshold	Sensitivity (%)	Specificity (%)	Accuracy (%)	PPV (%)	NPV (%)	AUC
Grayscale US (n=466)	type I-III vs IV	23.5 (12/51) [12.8,37.5]	98.3 (408/415) [96.6,99.3]	90.1 (420/466) [87.1,92.7]	63.2 (12/19) [38.4,83.7]	91.3 (408/447) [88.3,93.7]	0.83 [0.79, 0.86]
Lymphatic US (n=466)	pattern I-II vs III	37.3 (19/51) [24.1,51.9]	96.4 (400/415) [94.1,98.0]	89.9 (419/466) [86.81,92.5]	55.9(19/34) [37.9,72.8]	92.6 (400/432) [89.7, 94.9]	0.79 [0.75, 0.83]
Nomogram combining lymphatic and grayscale US findings (n=466)	40%	33.3 (17/51) [20.8,47.9]	98.1 (407/415) [96.2,99.2]	91.0 (424/466) [88.0,93.4]	68.0 (17/25) [46.5,85.1]	92.3 (407/441) [89.4,94.6]	0.88 [0.85, 0.91]

Note.—Data in parentheses are numbers of patients used to calculate percentages. Data in brackets are 95% confidence intervals. The enhancement pattern on CE US: pattern I, homogeneous; pattern II, inhomogeneous; pattern III, no enhancement. The LN morphology on grayscale US: type I, thin cortex; type II, diffuse cortical thickening; type III, focal eccentric cortical thickening; type IV, absent hilum. PPV = positive predictive value, NPV = negative predictive value, AUC = area under the receiver operating characteristic curve.

Supplementary Table 2. Comparison of Contrast agent for predicting ≥3 Metastatic ALNs

Imaging Methods	Sensitivity (%)	Specificity (%)	Accuracy (%)	PPV (%)	NPV (%)	AUC
Sonovue (n=254)	30.0 (9/30) [14.7, 49.4]	99.1 (222/224) [96.8, 99.9]	90.9 (231/254) [86.7, 94.2]	81.8 (9/11) [48.2, 97.7]	91.4 (222/243) [87.1, 94.6]	0.88 [0.83, 0.92]
Sonazoid (n=212)	38.1 (8/21) [18.1, 61.6]	96.9 (185/191) [93.3, 98.8]	91.0 (193/212) [86.4, 94.5]	57.1 (8/14) [28.9, 82.3]	93.4 (185/198) [89.0, 96.5]	0.88 [0.83, 0.92]

Note.—Data in parentheses are numbers of patients used to calculate percentages. Data in brackets are 95% confidence intervals.

PPV = positive predictive value, NPV = negative predictive value, AUC = area under the receiver operating characteristic curve.

Supplementary Table 3. Analysis of US findings of SLN according to reader 1, reader 2 at Center 1, and consensus reading.

	Development cohort (n=179)			Validation cohort (n=90)		
	Reader 1	Reader 2	Consensus reading	Reader 1	Reader 2	Consensus reading
**Enhancement patterns**						
Homogeneous enhancement, n (%)	43 (24)	46 (26)	42 (24)	13 (14)	16 (18)	17 (19)
Inhomogeneous enhancement, n (%)	120 (67)	118 (66)	122 (68)	69 (77)	65 (72)	65 (72)
No enhancement, n (%)	16 (9)	15 (8)	15 (8)	8 (9)	9 (10)	8 (9)
**Cortical morphology**						
Thin cortex, n (%)	86 (48)	87 (49)	88 (49)	54 (60)	56 (62)	57 (63)
Diffuse cortical thickening, n (%)	42 (24)	38 (21)	39 (22)	16 (18)	14 (16)	14 (16)
Focal eccentric cortical thickening, n (%)	45 (25)	49 (27)	46 (26)	19 (21)	18 (20)	17 (19)
Absent hilum, n (%)	6 (3)	5 (3)	6 (3)	1 (1)	2 (2)	2 (2)

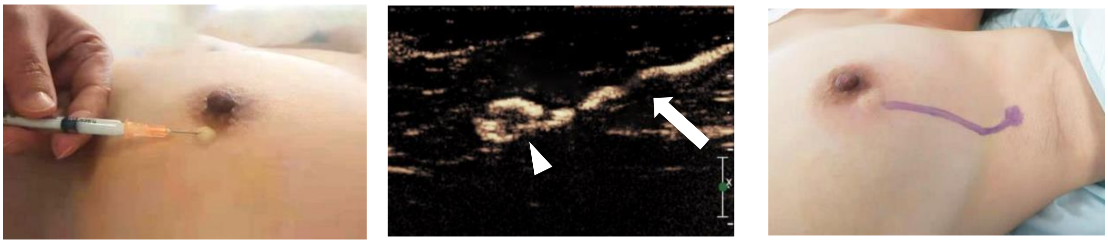

A B C

Supplementary Figure 1. Images in a 30-year-old woman with invasive ductal carcinoma in the left breast. **A.** Image of contrast agent injected intradermally into the periareolar area of the affected breast. **B.** The CEUS image of SLN. The SLN (arrowhead) was visualized along the enhanced lymphatic channel (arrow) and showed inhomogeneous pattern. **C.** The lymphatic channel (arrow) and location of the SLN (arrowhead) was marked on the skin. CEUS = contrast-enhanced ultrasound, SLN = sentinel lymph node.

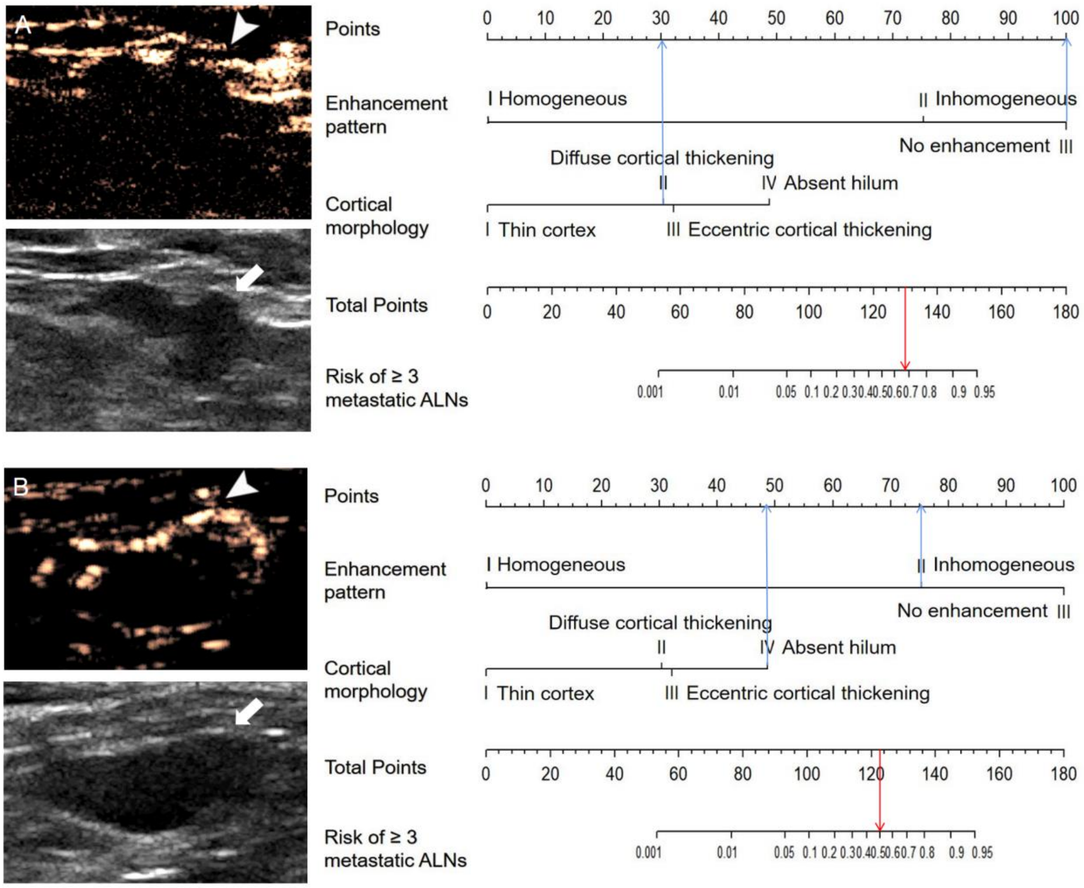

Supplementary Figure 2. Two false positive examples of the nomogram in clinical practice. **A.** The lymphatic and grayscale US findings of SLN in a 53-year-old woman with clinical T2 invasive carcinoma and 2 SLNs metastasis. The SLN showed no enhancement pattern and the cortical morphology of concentric thickening (arrows). An arrowhead indicates the enhanced lymphatic vessels. The total score is 130, which corresponds to about a 67.5% probability of having ⩾3 metastatic SLNs**. B.** The lymphatic and grayscale US findings of SLN in a 60-year-old woman with clinical T2 invasive carcinoma and 1 metastatic SLN. The SLN showed an inhomogeneous enhancement pattern and the absent hilum (arrows). An arrowhead indicates the enhanced lymphatic vessels. The total score is about 123, which corresponds to a 50 % probability of having ⩾3 metastatic ALNs.

ALN = axillary lymph node, SLN = sentinel lymph node.

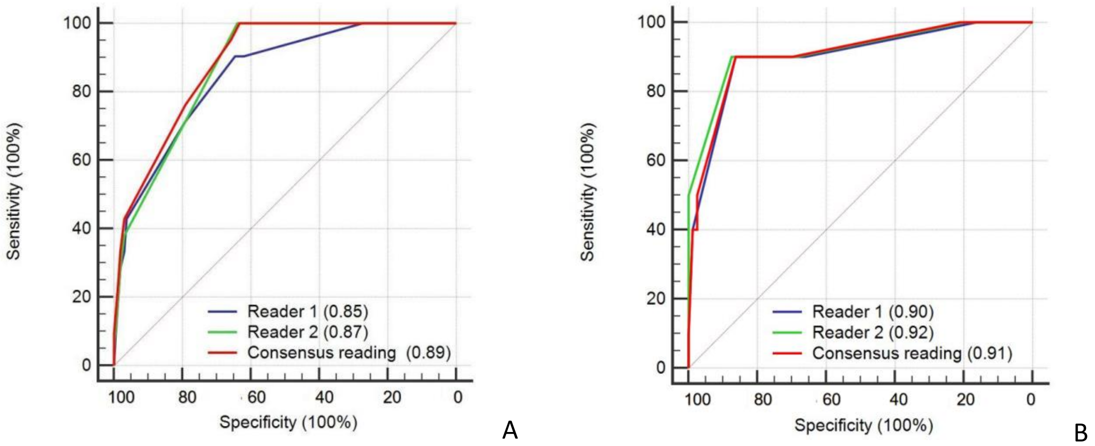

Supplementary Figure 3. Area under the curve (AUC) for predicting ALN disease burden of ≥3 metastatic ALNs according to reader 1, reader 2 and consensus reading at Center 1. **A.** AUC in development cohort. AUC of was 0.85 (95% CI: 0.79, 0.90), 0.87 (95% CI: 0.82, 0.92) and 0.89 (95% CI: 0.83, 0.93) for reader 1, reader 2 and consensus reading respectively. No statistically significant difference was found between the two readers (DeLong test, $p = 0.246$). **B.** AUC in validation cohort. AUC was 0.90 (CI 95%: 0.82, 0.96), 0.92 (95% CI: 0.84, 0.97) and 0.91 (95% CI: 0.82, 0.96) for reader 1, reader 2 and consensus reading respectively. No statistically significant difference were found between the two readers (DeLong test, $p = 0.235$). AUC = Area under the curve, ALN = axillary lymph node.
